# Supplementary material for: Cbfβ Is a Novel Modulator against Osteoarthritis by Maintaining Articular Cartilage Homeostasis through TGF-β Signaling
Source: Cells. 2023 Mar 31;12(7):1064. doi: 10.3390/cells12071064 (PMC10093452; doi:10.3390/cells12071064)
Supplement: Supplementary file 1 [file cells-12-01064-s001.zip › cells-2250454-supplementary.pdf]

a

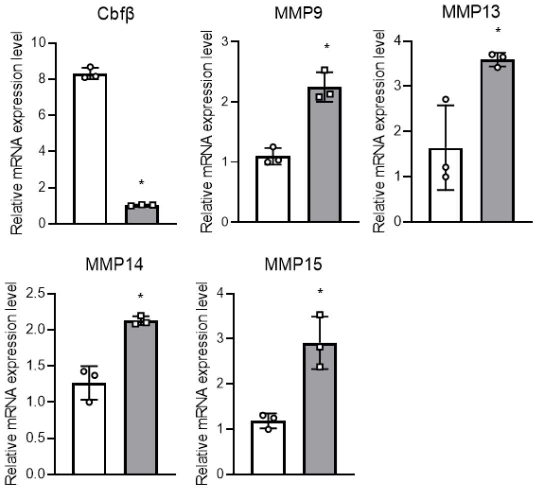

b

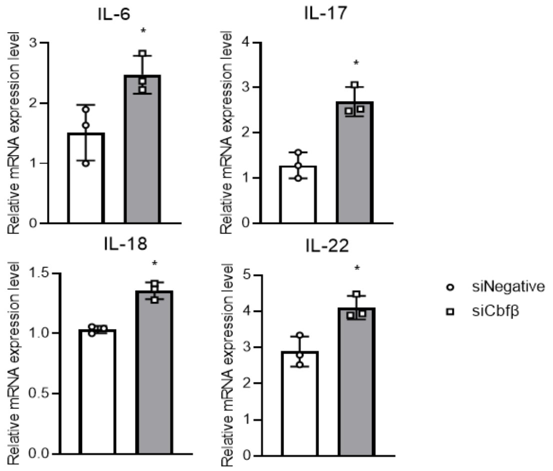

Figure S1. Cbfβ loss induces catabolic factors expression. (a and b) Mmps and inflammatory cytokine expression were performed by qRT-PCR.

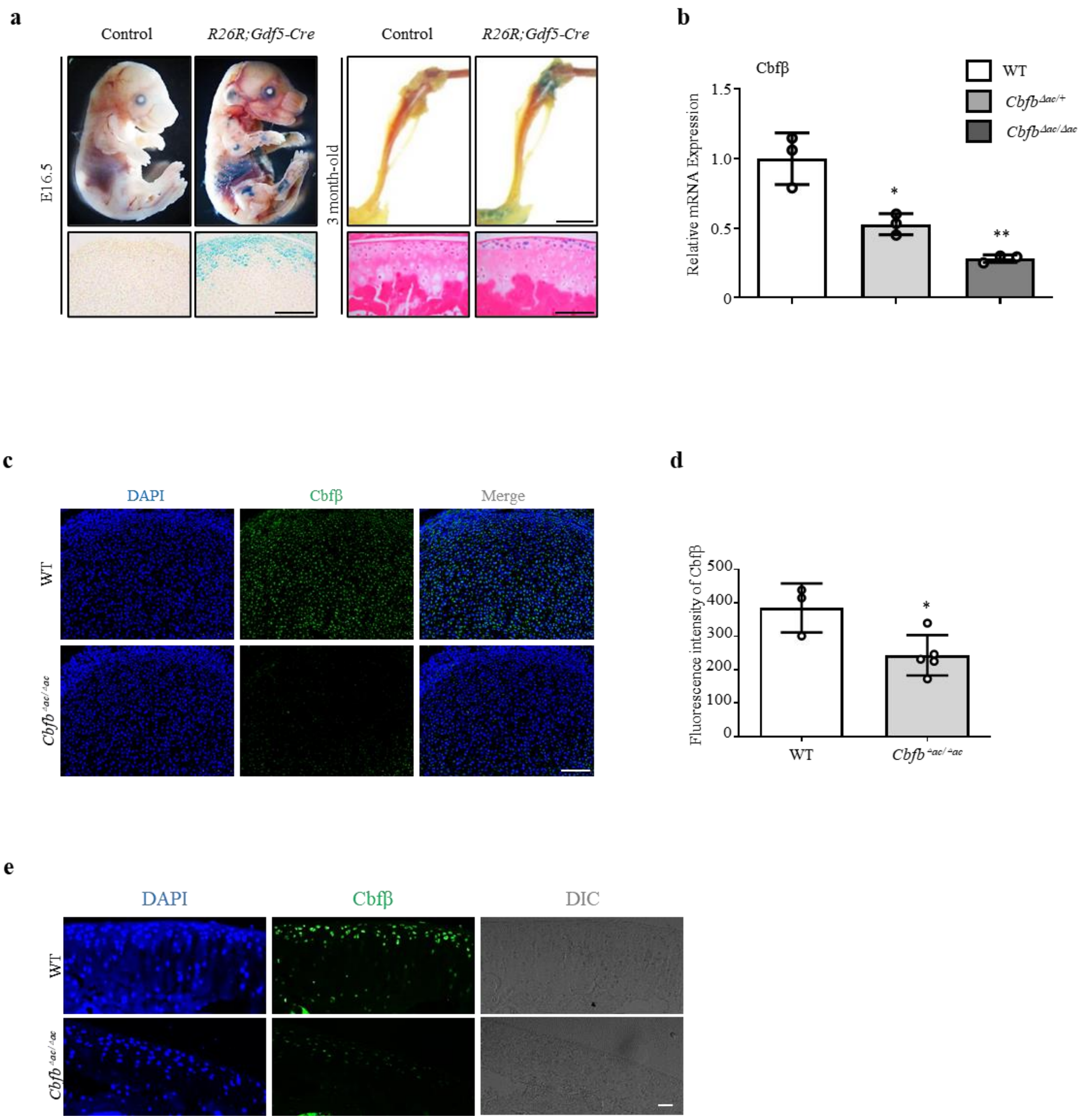

Figure S2. The Gdf5-Cre transgene is active in articular cartilage. (a) X-gal stained whole embryos and paraffin sections of the humerus from *R26R;Gdf5-Cre* and WT mice at embryonic day 16.5 (E16.5) (left panel). X-gal stained hindlimb (blue areas) and paraffin sections of the tibia joint from *R26R;Gdf5-Cre* and WT mice at 3 months (right panel). (b) The expression of Cbfβ in the $Cbfb^{\Delta ac/\Delta ac}$ articular chondrocytes was determined by qRT-PCR. (c) The expression of Cbfβ in articular chondrocytes at E16.5 was imaged by immunofluorescent staining, and its quantification was evaluated using Image J (d). (e) The expression of Cbfβ in articular chondrocytes at 20-week-old mice were imaged by immunofluorescent staining. DIC, differential interference contrast.
